# Imaging Scores in Subarachnoid Hemorrhage: Performance on Prediction of Functional Outcome, Mortality, and Complications

**DOI:** 10.3390/brainsci16010028

**Published:** 2025-12-25

**Authors:** Luise Biburger, Lena Mers, Anna Bogdanova, Alexander Sekita, Matthias Borutta, Daniel Delev, Yavor Bozhkov, Oliver Schnell, Tobias Engelhorn, Ludwig Singer, Maximilian Sprügel, Stefan Schwab, Stefan T. Gerner

**Affiliations:** 1 Department of Neurology, University of Erlangen-Nuremberg, 91054 Erlangen, Germany; 2 Department of Neurosurgery, University of Erlangen-Nuremberg, 91054 Erlangen, Germanyyavor.bozhkov@uk-erlangen.de (Y.B.);; 3 Department of Neuroradiology, University of Erlangen-Nuremberg, 91054 Erlangen, Germany

**Keywords:** subarachnoid hemorrhage, intraventricular hemorrhage, imaging scores, prognosis, functional outcome, ROC analysis

## Abstract

**Background/Objectives:** Several imaging scores have been developed for subarachnoid hemorrhage (SAH), but their prognostic performance for long-term functional outcome and post-hospital complications remains insufficiently characterized. We evaluated whether five admission imaging scores (modified Fisher, Claassen, Hijdra, Graeb, IVH) independently predict 12-month functional outcome and major secondary endpoints. **Methods:** We performed a retrospective cohort study of 479 consecutive patients with atraumatic SAH recorded in a prospectively maintained institutional registry. Admission CT/MRI was scored by two board-certified neuroradiologists blinded to clinical outcomes. The primary endpoint was unfavorable functional outcome at 12 months (modified Rankin scale [mRS] 4–6). Secondary endpoints included 12-month mortality, delayed cerebral ischemia (DCI), post-hemorrhagic epilepsy, shunt-dependent hydrocephalus, return to work, and patient-reported health. Receiver operating characteristic (ROC) analyses and multivariable logistic regression adjusted for established predictors were conducted. **Results:** All imaging scores were significantly associated with the primary endpoint and demonstrated adequate discrimination (area under the curve [AUC] ~0.70–0.74), with the Graeb and IVH scores performing highest for long-term functional outcome, mortality, and shunt dependence. Associations with DCI and epilepsy were modest. In multivariable analyses, all imaging scores remained independently associated with mRS 4–6. Subgroup analyses showed stronger prognostic performance in good-grade SAH, aneurysmal SAH, and cases with concomitant intraventricular hemorrhage. **Conclusions:** Admission imaging burden independently predicts 12-month functional outcome, mortality, and shunt dependence after SAH. Incorporating IVH-oriented measures alongside established clinical grading may improve individualized risk stratification, particularly in good-grade and aneurysmal SAH.

## 1. Introduction

Subarachnoid hemorrhage (SAH) remains a severe form of stroke associated with substantial long-term morbidity, particularly among relatively young patients [1]. Established clinical grading systems such as the World Federation of Neurosurgical Societies (WFNSs) scale and the Hunt and Hess score are routinely used to estimate early neurological severity, yet their ability to forecast long-term functional recovery and post-hospital complications remains limited. While aneurysmal rebleeding and delayed cerebral ischemia (DCI) are recognized determinants of poor outcome, long-term trajectories frequently extend beyond these early complications, underscoring the need for more comprehensive prognostic assessment [2].

Several imaging-derived scoring systems have been proposed to quantify acute hemorrhage burden in SAH, including the modified Fisher, Claassen, Hijdra, Graeb, and intraventricular hemorrhage (IVH) scores. However, most were primarily developed with respect to DCI risk, and their prognostic performance for extended functional outcome, mortality, return to work, or other late complications has not been systematically validated in large cohorts. Moreover, the prognostic relevance of admission imaging across meaningful clinical subgroups—such as good- versus poor-grade SAH, aneurysmal versus non-aneurysmal etiology, and the presence of concomitant IVH—remains insufficiently characterized.

The present study aimed to evaluate the prognostic value of five established SAH imaging scores for 12-month functional outcome and key secondary endpoints, including mortality and long-term complications. We further examined their performance across clinically relevant subgroups. We hypothesized that admission imaging burden would be independently associated with long-term outcomes and that IVH-oriented scores may provide incremental prognostic value in selected SAH populations.

## 2. Methods

We conducted a retrospective cohort study nested within a prospectively maintained institutional registry of patients with spontaneous (atraumatic) subarachnoid hemorrhage (SAH) admitted to the Departments of Neurology or Neurosurgery, University Hospital Erlangen, Germany, over a five-year period. This study was approved by the local ethics committee of the Friedrich-Alexander University Erlangen–Nuremberg (ethics vote: IRB reference 10_15 B). All patients or, where applicable, their legal representatives provided informed consent for data collection and analysis.

### 2.1. Patient Identification and Inclusion Criteria

All consecutive patients with spontaneous SAH were identified from the institutional database. Patients with secondary SAH due to trauma, intracerebral hemorrhage, or hemorrhagic transformation of ischemic stroke were excluded, as previously described [3].

### 2.2. Clinical Parameters

Demographic data (age and sex), prior medical history (i.e., premorbid score on the modified Rankin scale [mRS], prior medical history including arterial hypertension, and prior SAH), and clinical status on admission (Glasgow Coma Scale [GCS], Hunt and Hess [H&H], and World Federation of Neurosurgical Societies [WFNSs]) [4,5,6] were collected for each patient by medical record review. Patients were treated according to international and local guidelines [7]. In-hospital events, including documented delayed cerebral ischemia (DCI) and rebleeding, were prospectively recorded.

### 2.3. Imaging

SAH was diagnosed by CT imaging (SIEMENS Somatom Voume Zoom, Somatom Sensation 64, Somatom Definition AS+, Erlangen, Germany) or magnetic resonance tomography (SIEMENS Magnetom Sonata 1.5T, Magnetom Aera 1.5, Erlangen, Germany) on admission. Aneurysmal bleeding was either detected by CT-angiography or digital subtraction angiography [DSA].

The initial imaging leading to the diagnosis of SAH was used for assessment of all imaging scores. The extent of SAH was scored by two blinded neuroradiologists using the modified Fisher [8], Claassen [9], and Hijdra [10] scores—see Table 1 for further details. In addition, IVH was measured using the Graeb [11] and IVH scores [12] (calculation shown in Table 1). Disagreements were resolved by consensus review.

In follow-up intracranial imaging, the occurrence of rebleeding and delayed cerebral ischemia [DCI] was scored according to current definitions [13]. Any new infarction during follow-up imaging attributable to vasospasm was defined as DCI [13].

### 2.4. Outcome Measures

Long-term outcomes were assessed at 12 months after initial SAH, as described previously [3]. Long-term outcomes included functional status assessed using the mRS, mortality, presence of post-hemorrhagic epilepsy, need for permanent CSF shunting due to chronic hydrocephalus, return to work, and self-reported quality of health using the EQ-5D (www.euroqol.org; [14]). Follow-up assessments were conducted after 12 months by either mailed questionnaires or semi-structured telephone interviews by physicians certified for stroke outcome assessments (LB, STG).

Primary endpoint was unfavorable functional outcome, defined as an mRS score (ranging from 0, no deficit, to 6, death) of 4 or higher. Secondary endpoints comprised mortality at 12 months, occurrence of DCI during hospital stay, and long-term complications, including development of post-hemorrhagic epilepsy as well as requirement for CSF shunting due to chronic hydrocephalus [15] after 12 months. In a subgroup of patients younger than 65 years and employed at the time of SAH, return to prior work was recorded. Quality-of-life was measured by the standardized EQ-5D-3L tool [14]. Patients were asked to graduate their health status in five dimensions, including mobility, self-care, usual activities, pain/discomfort, and anxiety or depression, and to rate their overall health status using a visual analog scale (VAS) in the questionnaire or a numerical analog scale during the phone interview at follow-up. A VAS score (ranging from 0 to 100) of 75 to 100 was defined as good subjective health.

### 2.5. Statistical Analyses

Statistical analyses were performed using SPSS v24.0 (IBM Corp., Armonk, NY, USA). Receiver operating characteristic (ROC) analyses were used to determine the discriminative ability of admission imaging scores for the primary endpoint (mRS 4–6 at 12 months) and secondary outcomes. Results were presented as the area under the ROC curve (AUC) with corresponding 95% confidence intervals (CIs), using the admission WFNS grade as a clinical reference. AUC values were classified as follows: 1.0 = perfect test, 0.90–0.99 = excellent, 0.80–0.89 = good, 0.70–0.79 = adequate, and <0.70 = poor [16]. Additional analyses by multivariable modeling were conducted to address potential bias and find independent associations between the investigated imaging scores and endpoints after adjustment for already-established predictors of each outcome [2]; see the legend of the corresponding table for further details. All associations were presented as adjusted odds ratios (aORs) with corresponding 95% confidence intervals (95% CIs) in multivariable analyses. Subgroup analyses were prespecified for good-grade (WFNS 1–3) versus poor-grade (WFNS 4–5) SAH, aneurysmal versus non-aneurysmal SAH, and presence versus absence of concomitant IVH. Statistical significance was defined as *p* < 0.05. Analyses were performed using complete-case data; no imputation for missing values was applied.

## 3. Results

**Figure 1:**
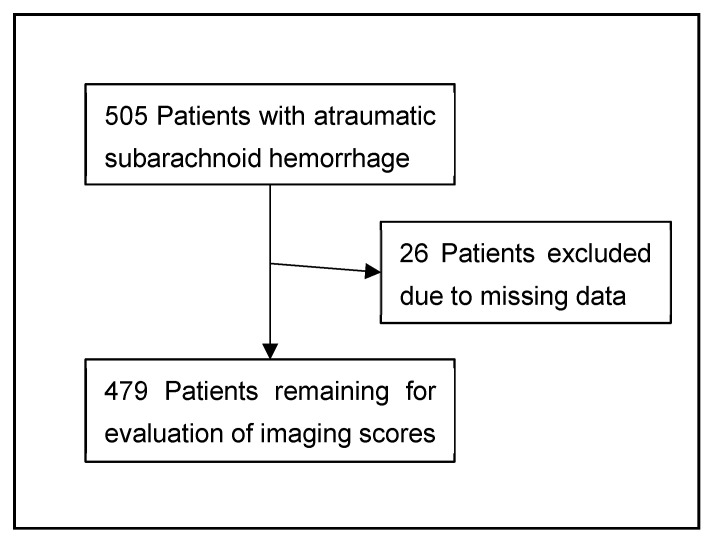
Flowchart of patients. Over a five-year period, overall 505 patients with atraumatic subarachnoid hemorrhage were included in the institutional database. After exclusion of those with missing imaging data (*n* = 26), 479 SAH patients were available for final analyses.

Overall, 505 patients with atraumatic SAH over a five-year period were included in the institutional database. After exclusion of patients without initial imaging available (*n* = 26), 479 patients remained for final analyses (see Figure 1).

The mean age (SD) was 56.1 (13.6) years, and 307 (64.1%) patients were female (Table 2). Median GCS (interquartile range) on admission was 14 (11–15), median Hunt and Hess was 2 (1–4), and median WFNS grade was 2 (2–4). A total of 298 (62.5%) patients were defined as good-grade SAH (WFNS 1–3) and 179 (37.5%) as poor-grade SAH (WFNS 4–5). Among patients with vascular imaging (*n* = 466), aneurysmal SAH was identified in 75.4%. Concomitant IVH was present in 59.9% of the cohort.

### 3.1. Imaging Assessment on Admission

Median imaging scores on admission were as follows: modified Fisher 2 (IQR 1–3), Claassen 3 (IQR 2–4), Hijdra 8 (IQR 4–8), Graeb 1 (IQR 1–3), and IVH score 4 (IQR 4–9) (Table 2). A total of 56 (11.6%) patients had a Graeb score ≥ 5, indicating substantial IVH.

### 3.2. Outcomes

After 12 months, the median mRS (IQR) was 2 (1–4), and the rate of unfavorable outcome—defined as mRS 4–6—was 28% (Table 2). The mortality rate was 19.6% (94/479). DCI occurred in 134 (28.4%) patients during hospital stay. Rates for long-term complications were 10.2% (*n* = 34) for post-hemorrhagic epilepsy and 10.2% (*n* = 99) for chronic hydrocephalus requiring permanent CSF drainage.

During follow-up, 137/236 (58.1%) patients of working age returned to work. Overall, patient-reported health was stated in median (IQR) at 75 (VAS). Problems in the domains of mobility/self-care/usual activities/pain/anxiety were reported to be absent in 19.9%/18.1%/34.6%/36.9%/47.9%, respectively.

### 3.3. ROC Analyses of Initial Imaging with the Primary Endpoint

**Figure 2:**
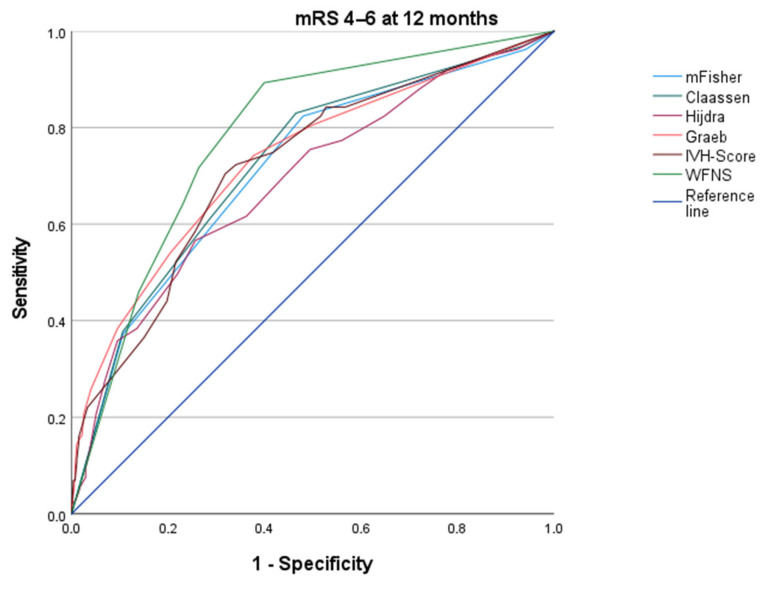
ROC curve of imaging scores and primary endpoint (mRS at 12 months). Receiver operating characteristic (ROC) analysis for the association between imaging scores (modified Fisher, Claassen, Hijdra, Graeb, IVH score) and mRS (4–6) at 12 months, with WFNS as the clinical comparator. The reference line is illustrated in dark blue.

ROC curves illustrating the association between imaging parameters and unfavorable functional outcome at 12 months are shown in Figure 2. Significant associations (*p* < 0.05) were found for all tested imaging scores and the clinical comparator (WFNS on admission).

In the overall cohort, adequate discrimination (i.e., AUROC 0.7–0.79 and CI defined as 95% confidence interval) for unfavorable outcome was achieved by modified Fisher (AUROC 0.713; CI 0.662–0.764), Claassen (AUROC 0.724; CI 0.675–0.774), Graeb (AUROC 0.732; CI 0.682–0.782), and IVH score (AUROC 0.721; CI 0.671–0.771). Measurement of SAH extent by the Hijdra grading scale (AUROC 0.688; CI 0.635–0.740) had a poor association. Clinical grading by WNFS on admission showed adequate correlation with functional outcome (0.782; CI 0.738–0.827).

### 3.4. ROC Analyses of Secondary Endpoints

**Figure 3:**
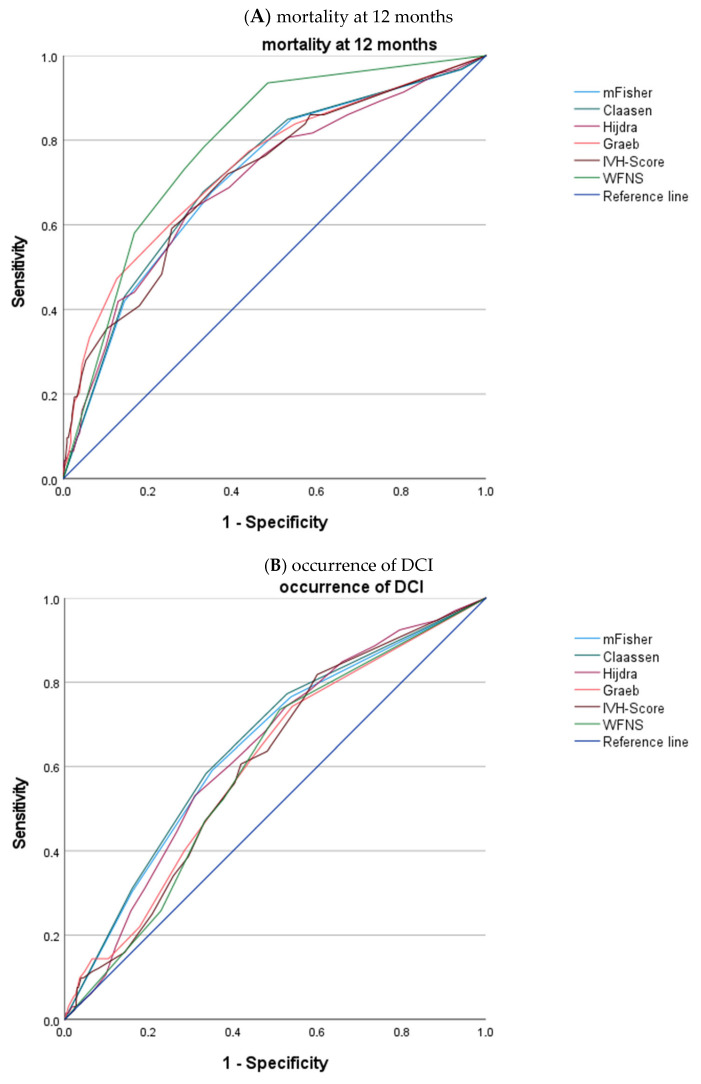

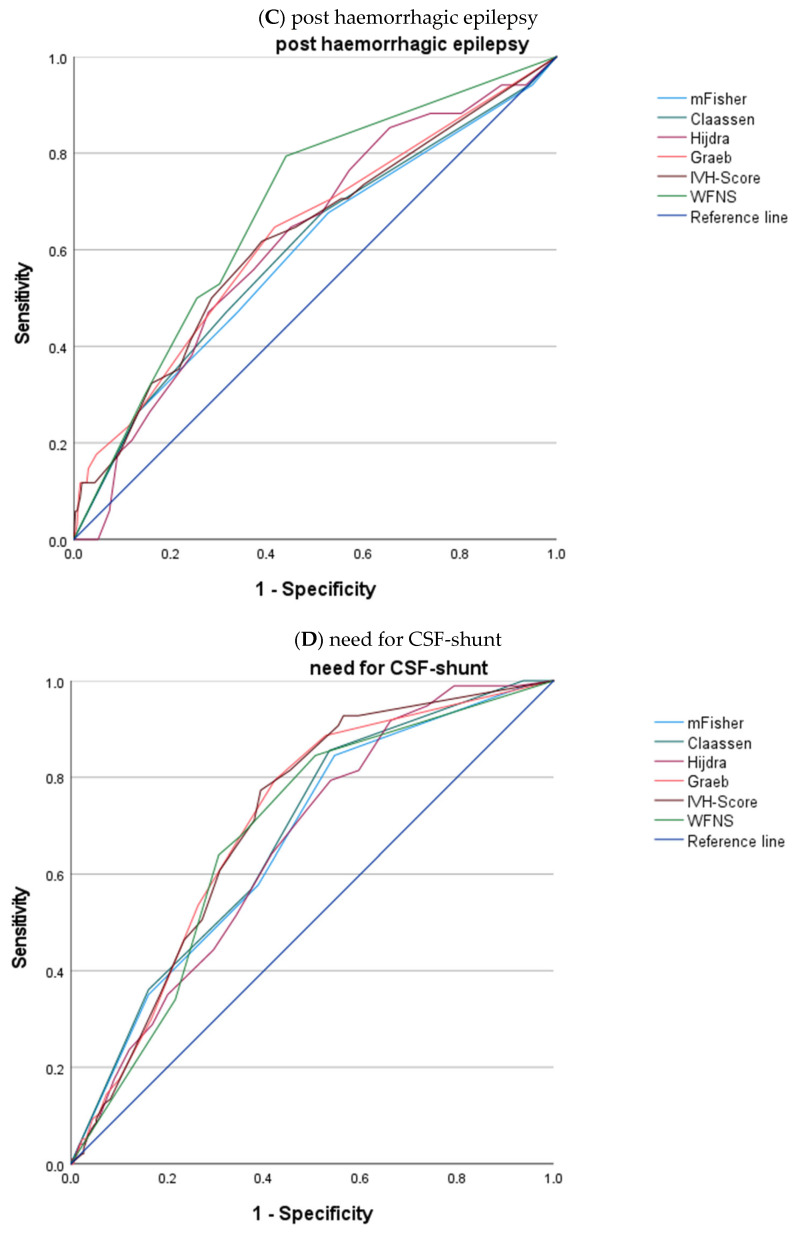
ROC curve of imaging scores and secondary endpoints. Receiver operating characteristic (ROC) analysis for the association between imaging scores (modified Fisher, Claassen, Hijdra, Graeb, IVH score) and secondary endpoints are as follows: (**A**) mortality at 12 months, (**B**) occurrence of DCI during hospital stay, (**C**) occurrence of post-hemorrhagic epilepsy within 12 months, and (**D**) need for CSF shunting within 12 months. WFNS served as the clinical comparator. The reference line is illustrated in dark blue.

Graphical regression analyses for our secondary endpoints are shown in Figure 3A. For 12-month mortality, all tested imaging scores were significantly correlated (*p* < 0.05), with the Hijdra score revealing the weakest correlation (AUROC 0.701, CI 0.639–0.763) and the Graeb score revealing the strongest correlation (AUROC 0.736, CI 0.676–0.796). Clinical WFNS had an AUROC of 0.791 (95%CI 0.743–0.839).

There was a significant but poor correlation between imaging scores and the occurrence of DCI and development of post-hemorrhagic epilepsy (see Figure 3B,C). For DCI, AUROC ranged from 0.605 (CI 0.549–0.660, Graeb) to 0.652 (CI 0.597–0.706), while the Hijdra score showed the weakest correlation, with AUROC values ranging from 0.621 (CI 0.525–0.716; Hijdra) to 0.683 (CI 0.594–0.773; WFNS) for epilepsy. The association between imaging scores and occurrence of chronic hydrocephalus was poor, with SAH extent measured by the Claassen scale showing the strongest (AUROC 0.679, CI 0.623–0.734) correlation (shown in Figure 3D).

### 3.5. Multivariable Analysis

In multivariate analysis, all imaging scores showed independent and significant associations with the primary endpoint (mRS 4–6 at 12 months; Table 3). The adjusted odds ratios (aORs) for unfavorable functional outcome (mRS 4–6) per one-point increase in imaging score were as follows (Table 3): 1.060 (95% CI 1.022–1.101) for the modified Fisher score, 1.070 (95% CI 1.030–1.110) for the Claassen score, 1.009 (95% CI 1.001–1.018) for the Hijdra score, 1.046 (95% CI 1.028–1.065) for the Graeb score, and 1.018 (95% CI 1.010–1.026) for the IVH score, respectively. For the secondary endpoints, independent associations with mortality at 12 months and CSF shunting were found for all tested imaging scores. Only the modified Fisher (OR 1.084, CI 1.043–1.136) and Claassen (OR 1.090, CI 1.049–1.132) scores were independently associated with the occurrence of DCI, whereas the other imaging scores did not reach statistical significance after adjustment for covariates.

### 3.6. Subgroup Analyses Regarding the Primary Endpoint

**Figure 4:**
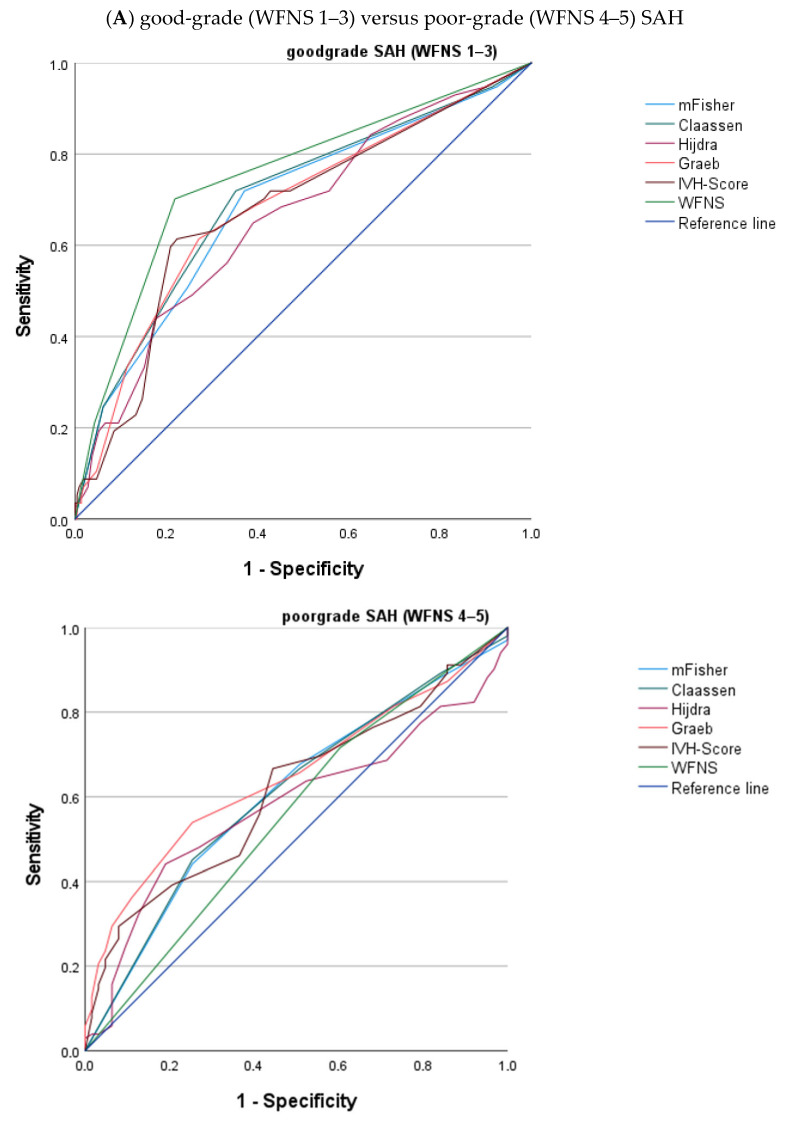

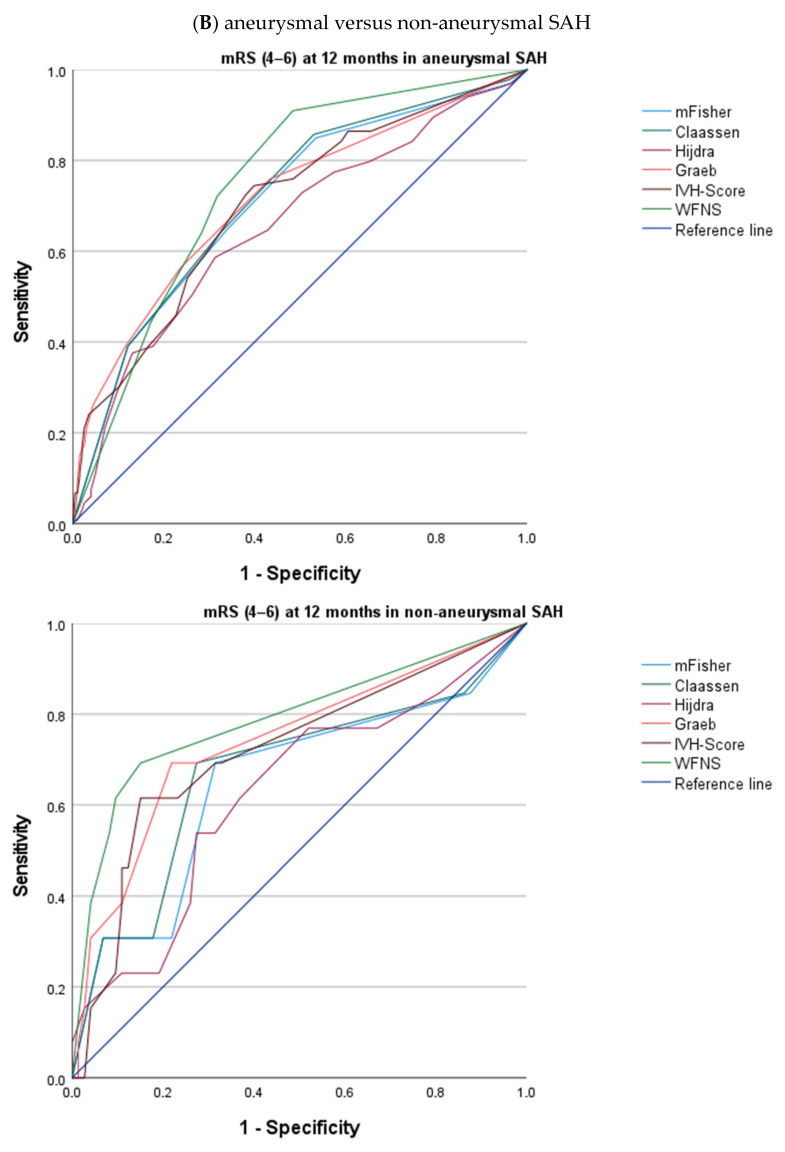

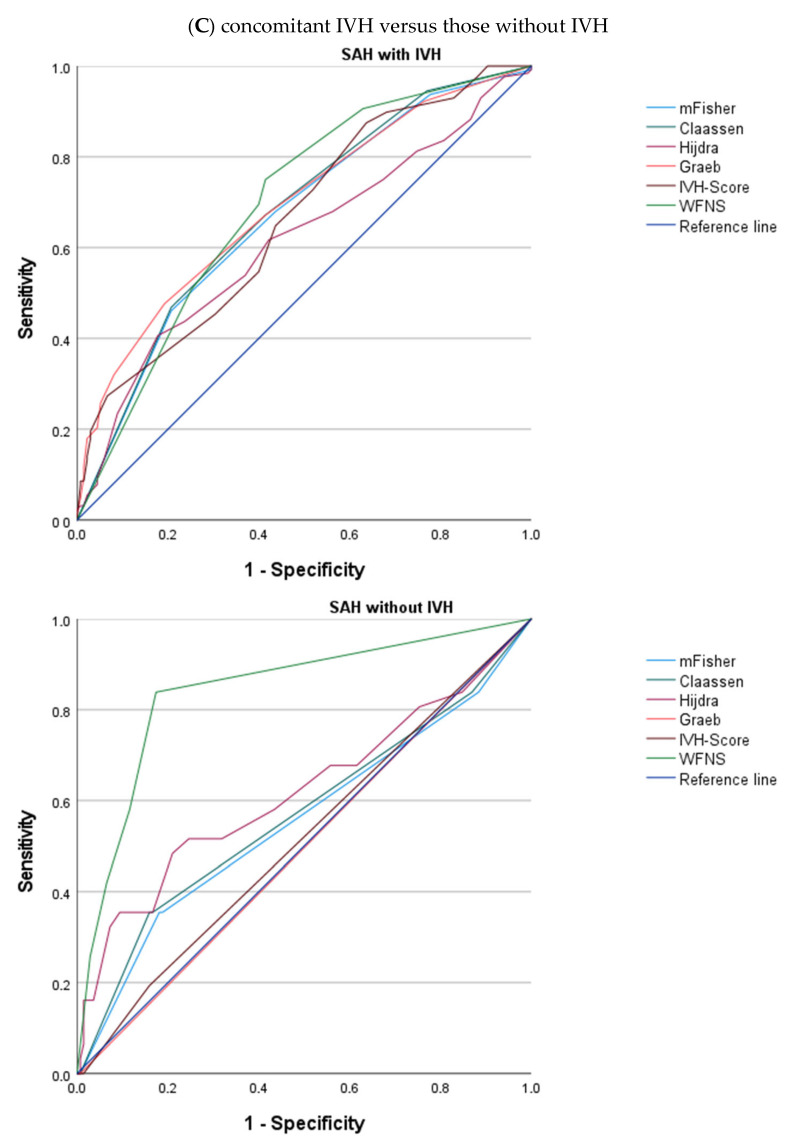
ROC curve of imaging scores and mRS at 12 months, dichotomized into three groups. Receiver operating characteristic (ROC) analyses for the following subgroups: (**A**) good-grade (WFNS 1–3) versus poor-grade (WFNS 4–5) SAH, (**B**) aneurysmal versus non-aneurysmal SAH, and (**C**) SAH patients with versus without concomitant IVH. ROC analyses show the association between imaging scores (modified Fisher, Claassen, Hijdra, Graeb, IVH score) and unfavorable outcome (i.e., mRS = 4–6) at 12 months. WFNS served as the clinical comparator. The reference line is illustrated in dark blue.

Subgroup analyses were performed for the following groups: (a) good-grade (WFNS 1–3) versus poor-grade (WFNS 4–5) SAH, (b) aneurysmal vs. non-aneurysmal SAH, and (c) SAH with versus without concomitant IVH (for further details, see Figure 4A–C).

After classification of patients into good-grade (WFNS 1–3) and poor-grade (WFNS 4–5) SAH, correlations between imaging scores and the primary endpoint remained significant (*p* < 0.05) (Figure 4A). In good-grade SAH, there were better associations between imaging scores and unfavorable outcome than in patients with poor-grade SAH (e.g., modified Fisher in good-grade SAH: AUROC 0.703, CI 0.609–0.797; modified Fisher in poor-grade SAH: AUROC 0.613, CI 0.530–0.696). Correlation between imaging scores and poor outcome (mRS 4–6) was significant (*p* < 0.05) for all imaging scores in both subgroups. Adequate AUROC values (≥0.7) were reached by modified Fisher (AUROC 0.703, CI 0.609–0.797) and Claassen (AUROC 0.713, CI 0.619–0.807) only in the subgroup of good-grade (WFNS 1–3) patients.

Subanalysis regarding aneurysmal SAH showed similar correlations to the main analysis (Figure 4B). In the aneurysmal subgroup, adequate AUROC (0.7–0.79) values were reached by modified Fisher (AUROC 0.728, CI 0.672–0.785), Claassen (AUROC 0.732, CI 0.675–0.788), and Graeb (AUROC 0.716, CI 0.633–0.754). In non-aneurysmal SAH, there was no significant correlation between imaging scales and functional outcome, but the analysis was limited by low patient numbers (*n* = 105).

Third, patients were classified by presence or absence of concomitant IVH (Figure 4C). In patients without IVH (*n* = 192), the only parameter significantly associated with the primary endpoint was WFNS on admission (AUROC 0.842, CI 0.759–0.925), whereas no imaging parameter showed good correlation. In patients with SAH and concomitant IVH, imaging parameters showed significant but poor correlation (AUC < 0.7) for mRS 4–6 at 12 months.

## 4. Discussion

In this study, five established imaging scores demonstrated significant associations with unfavorable functional outcome (mRS 4–6), mortality, and shunt dependence at 12 months following subarachnoid hemorrhage (SAH). Prognostic performance was most pronounced in patients with good-grade SAH, aneurysmal etiology, and concomitant intraventricular hemorrhage (IVH). These findings indicate that initial hemorrhage burden carries prognostic information extending beyond the prediction of delayed cerebral ischemia (DCI), particularly for long-term outcomes that are increasingly relevant in modern SAH care.

Up to the present, standardized assessment of imaging data in SAH is not well established, contrary to ICH, where imaging-based ICH characteristics represent a validated prognostic parameter included in established prognostic models [17]. So far, imaging scores in SAH have been mainly developed for prediction of DCI [18,19]. Their use for predicting outcomes other than DCI is not well established, and available data are limited by small patient samples, deficient outcome assessment beyond functional impairment, and short follow-up periods [20,21]. Our findings expand this knowledge by showing that imaging scores are not only associated with functional outcome and DCI, but also with post-hospital complications and patient-reported outcomes.

Across all investigated outcomes, the imaging scores correlated significantly with functional outcome, mortality, DCI, post-hemorrhagic epilepsy, CSF shunt dependency, return to work, and quality-of-life (EQ-5D). Although the strength of association varied between endpoints, the overall positive correlation highlights the prognostic value of initial hemorrhage burden in SAH. The strongest associations were found for long-term functional outcome at 12 months. Most prior studies focused on shorter follow-up intervals (usually three months), despite evidence that recovery after SAH frequently extends beyond this time frame [22]. Associations with EQ-5D outcomes were more modest, consistent with the multidimensional nature of subjective health and the limited capacity of acute imaging to capture chronic symptoms such as persistent headache [3].

Need for permanent CSF drainage is a frequent and severe complication after SAH and increases patient morbidity and readmission rates [23,24]. Several risk factors for the need for a permanent CSF shunt have already been identified, including intraventricular hemorrhage [25]. In accordance, imaging scores measuring the extent of IVH (i.e., Graeb and IVH score) showed better correlation than scores focusing on SAH extent with long-term shunt dependence in our analysis. This aspect indicates the importance of IVH assessment for the prediction of permanent CSF shunting. Epilepsy after SAH occurs in 7% to 12% of patients within 12 months and is associated with higher morbidity [26]. The pathophysiological mechanism has not yet been established, but several risk factors are known, such as the presence of additional IVH [27]. In our analysis, post-hemorrhagic epilepsy correlated significantly with IVH scores (i.e., Graeb, IVH score), which supports this assumption. Furthermore, the extent of SAH in basal cisterns measured by the Hijdra score seems to have further impact on the occurrence of epilepsy—an aspect which needs further attention in future trials.

Return to work and quality-of-life are increasingly recognized as relevant endpoints beyond traditional functional scales. Although imaging burden was associated with both domains, the magnitude of these associations was modest, indicating that late recovery and social reintegration are determined by clinical, psychosocial, and demographic factors that extend beyond initial radiographic findings. These observations underscore the need for multidimensional prognostic approaches in SAH research [28].

In our subgroup analysis, there were three major findings. First, in the good-grade (WFNS 1–3) vs. poor-grade (WFNS 4–5) subgroup, correlations between imaging scores and outcome were weaker in the latter, which presented with poor clinical condition on arrival. According to an analysis by Manoel et al. [29], one-third of poor-grade SAH patients achieve a good functional outcome, whereas one-fifth of good-grade SAH patients have a poor functional outcome [30]. Our study suggests that the importance of imaging characteristics is pronounced in patients presenting with good clinical condition on arrival at a lower risk for DCI and hydrocephalus, whereas in poor-grade patients, prediction of functional outcomes is limited due to the higher rate of serious complications contributing to worse morbidity and increased mortality, which is not reflected by imaging alone.

Second, imaging scores correlated with outcomes only in aneurysmal SAH, but not in non-aneurysmal SAH, likely reflecting differences in underlying pathophysiology and the fact that most imaging scores were originally validated in aneurysmal cases. Third, prognostic accuracy was highest in patients with concomitant IVH, reinforcing the notion that intraventricular blood burden is a major determinant of long-term outcome.

An important question is how imaging scores complement established clinical grading systems such as the WFNS score. Since its introduction in 1988, substantial advances have occurred in SAH management [31]. Our results suggest that incorporating IVH-oriented measures may refine individualized risk estimation in selected SAH populations. Although clinical grading systems such as the WFNS score remain the cornerstone of early prognostication, imaging-based parameters provide complementary information and may serve as pragmatic and readily available markers to inform discussions with patients and families, particularly regarding long-term expectations and risk of permanent CSF diversion. Although combined clinical–imaging models have been proposed [32,33,34], none have yet demonstrated superiority over WFNS in routine clinical practice. The present study provides a systematic comparison of conventional imaging scores and their relevance for long-term outcomes, supporting the inclusion of imaging variables into future multimodal prediction models [35].

This study has limitations. The retrospective design introduces potential selection bias, despite the use of a prospectively maintained database. Follow-up assessments involving mailed questionnaires and telephone interviews may be subject to reporting bias, and longer observation periods might be necessary to fully capture quality-of-life trajectories. Use of multiple CT/MRI scanners might have introduced heterogeneity, though all scores rely on qualitative assessment of hemorrhage burden. Individual rater-level datasets were not archived, precluding formal calculation of inter-rater reliability; consensus-based scoring was employed to ensure uniformity. Finally, despite adjustment for established predictors, residual confounding cannot be excluded, and as this was a single-center study conducted at a tertiary care hospital, the generalizability of our findings may be limited.

## 5. Conclusions

In conclusion, admission imaging burden is independently associated with long-term functional outcome, mortality, and shunt dependence after SAH, with enhanced prognostic performance in selected patient subgroups. These findings support the integration of imaging-derived measures into future multimodal prognostic models and reinforce the relevance of structured radiographic assessment at admission.

## Figures and Tables

**Table 1:** Summary of imaging scores used for measurement of SAH and IVH.

	SAH-Scores			IVH Scores	
	Modified Fisher Scale	Claassen Score	Hijdra Score	Graeb Score	IVH Score
**Publication date**	2001	2001	1990	1982	2009
**Range**	0–4	1–5	0–42	0–12	0–23
**Score definition/calculation**	0: No SAH or IVH 1: Thin SAH, no IVH in both lateral ventricles 2: Thin SAH, with IVH in both lateral ventricles 3: Thick SAH, no IVH in both lateral ventricles 4: Thick SAH, with IVH in both lateral ventricles	1: No SAH or IVH 2: Thin SAH, no IVH in both lateral ventricles 3: Thin SAH, with IVH in both lateral ventricles 4: Thick SAH, no IVH in both lateral ventricles 5: Thick SAH, with IVH in both lateral ventricles	10 basal cisterns (0–3): 0: no blood 1: small amount of blood 2: moderately filled with blood 3: completely filled with blood Each ventricle (0–3): 0: no blood 1: sedimentation of blood 2: partly filled with blood 3: completely filled with blood	Each lateral ventricles: 1: trace of blood or mild bleeding 2: less than half of the ventricle filled with blood 3: more than half of the ventricle filled with blood 4: ventricle filled with blood and expanded Third and fourth ventricles: 0: no blood 1: blood present, ventricle size normal 2: ventricle filled with blood and expanded	**IVHS = 3x (RV + LV) + III + IV+ 3x H** Grade each lateral ventricle (RV/LV): 0: no blood or small amount 1: up to one-third filled with blood 2: one-to-two-thirds filled with blood 3: mostly or completely filled with blood Third and fourth ventricle (III/IV): 0: no blood 1: partially or completely filled with blood Hydrocephalus (H) present (1) or absent (0).
**Validated for:**					
- **Aneurysmatic SAH**	Yes	Yes	Yes	Yes	No
- **Non-aneurysmatic SAH**	Yes	Yes	No	Yes	No
- **Traumatic SAH**	No	No	No	Yes	No
- **DCI**	Yes	Yes	Yes	No	No
- **Mortality**	No	No	Yes	Yes	Yes
- **Functional outcome**	No	No	No	Yes	Yes

Summary of imaging scores for SAH and IVH with name, year of publication, range, definition, and previous validation. Abbreviations: SAH, subarachnoid hemorrhage; IVH, intraventricular hemorrhage; IVHS, IVH score; RV, right ventricle; LV, left ventricle; III, third ventricle; IV, fourth ventricle; H, hydrocephalus; DCI, delayed cerebral ischemia.

**Table 2:** Demographic and clinical characteristics of the included patients.

	SAH Patients (*n* = 479)
*Age, y, mean (SD)*	56.1 (±13.6)
*Female sex, n (%)*	307 (64.1)
***Prior comorbidities:***	
*Premorbid mRS, median (IQR)*	0 (0–0)
*Arterial hypertension, n (%)*	267 (55.7)
*Nicotine abuse, n (%)*	137 (28.6)
*Prior SAH, n (%)*	5 (1.0)
***Admission status:***	
*GCS, median (IQR)*	14 (11–15)
*Hunt and Hess, median (IQR)*	2 (1–4)
*WFNS, median (IQR)*	2 (2–4)
*Poor-grade SAH (WFNS ≥ 4), n (%)*	179/477 (37.5)
***SAH characteristics:***	
*Aneurysmal SAH, n (%)*	361/466 (75.4%)
*Intraventricular hemorrhage, n (%)*	287/479 (59.9%)
***Imaging scores:***	
***mFisher score, median (IQR)***	2 (1–3)
*0, n (%)*	23 (4.8)
*1 or 2, n (%)*	251 (52.4)
*3 or 4, n (%)*	205 (42.7)
***Claassen score, median (IQR)***	3 (2–4)
*1, n (%)*	24 (5)
*2 or 3, n (%)*	257 (53.7)
*4 or 5, n (%)*	197 (41.2)
***Hijdra score, median (IQR)***	8 (4–8)
*0–10, n (%)*	322 (67.2)
*11–20, n (%)*	150 (31.3)
*21–30, n (%)*	7 (1.4)
***Graeb score, median (IQR)***	1 (1–3)
*0–4, n (%)*	423 (88.3)
*5–8, n (%)*	39 (8.1)
*9–12, n (%)*	17 (3.5)
***IVH Score, median (IQR)***	4 (4–9)
*0–7, n (%)*	326 (68.0)
*8–15, n (%)*	125 (26.0)
*16–23, n (%)*	28 (5.8)
***Outcome:***	
*Doppler sonographic vasospasm n (%)*	182/402 (45.3)
*DCI, n (%)*	134/472 (28.4)
*CSF shunt, n (%)*	99 (20.7)
*Epilepsy, n (%)*	34/334 (10.2)
*Mortality at discharge*	69 (14.4)
*Mortality at 12-month, n (%)*	94 (19.6)
***mRS at discharge, median (IQR)***	3 (2–5)
*Favorable 0–3, n (%)*	248 (51.8)
*Unfavorable 4–6, n (%)*	231 (48.2)
***mRS at 3 months, median (IQR)***	2 (1–5)
*Favorable 0–3, n (%)*	275/415 (66.3)
*Unfavorable 4–6, n (%)*	140/415 (33.7)
***mRS at 12 months, median (IQR)***	2 (1–4)
*Favorable 0–3, n (%)*	313/435 (72.0)
*Unfavorable 4–6, n (%)*	122/435 (28.0)
*Return to work, n (%)*	137/236 (58.1%)
*EQ-VAS index, median (IQR)*	75 (55–90)
*Unfavorable 0–74, n (%)*	154/333 (46.2)
*Favorable 75–100, n (%)*	179/333 (53.8)
***EQ-5D domains:***	
*Mobility = 0, n (%)*	66/332 (19.9)
*Self-care = 0, n (%)*	60/332 (18.1)
*Usual activities = 0, n (%)*	115/332 (34.6)
*Pain/discomfort = 0, n (%)*	122/331 (36.9)
*Anxiety/depression = 0, n (%)*	158/330 (47.9)

Overall, 479 patients with SAH were recorded. Demographic and clinical characteristics, imaging data, and outcome parameters are presented. Dichotomous data are presented with *n* (%). Mean (SD) and median (IQR) are provided for normally and non-normally distributed parameters, respectively. Abbreviations: SD, standard deviation; IQR, interquartile range; SAH, subarachnoid hemorrhage; GCS, Glasgow Coma Scale; WFNS, World Federation of Neurosurgical Societies; DCI, delayed cerebral ischemia; CSF, cerebrospinal fluid; mRS, modified Rankin scale; EQ-5D, EuroQol 5 Dimensions; VAS, visual analog scale; IVH, intraventricular hemorrhage; y, years.

**Table 3:** Overview of AUROC values and adjusted odds ratios of imaging scores and outcomes.

Imaging Score	mRS 4–6 (12 m)		Mortality (12 m)		CSF Shunt		EQ-5D	
	**AUROC (95% CI)**	**Adj. OR (95% CI)**	**AUROC (95% CI)**	**Adj. OR (95% CI)**	**AUROC (95% CI)**	**Adj. OR (95% CI)**	**AUROC (95% CI)**	**Adj. OR (95% CI)**
Modified Fisher	**0.713 (0.662–0.764)**	**1.060 (1.022–1.101)**	**0.713 (0.654–0.772)**	**1.042 (1.009–1.076)**	**0.721 (0.661–0.780)**	**1.053 (1.018–1.089)**	**0.410 (0.349–0.471)**	0.968 (0.923–1.016)
Claassen	**0.724 (0.675–0.774)**	**1.070 (1.030–1.110)**	**0.721 (0.662–0.779)**	**1.045 (1.011–1.080)**	**0.736 (0.679–0.793)**	**1.058 (1.022–1.095)**	**0.402 (0.341–0.463)**	0.961 (0.915–1.009)
Hijdra	**0.688 (0.635–0.740)**	**1.009 (1.001–1.018)**	**0.702 (0.641–0.764)**	**1.009 (1.001–1.017)**	**0.740 (0.683–0.796)**	**1.010 (1.002–1.018)**	**0.422 (0.361–0.484)**	0.995 (0.983–1.008)
Graeb	**0.732 (0.682–0.782)**	**1.046 (1.028–1.065)**	**0.735 (0.675–0.794)**	**1.036 (1.016–1.056)**	**0.761 (0.703–0.819)**	**1.020 (1.001–1.041)**	**0.407 (0.346–0.468)**	**0.967 (0.944–0.991)**
IVH Score	**0.721 (0.671–0.771)**	**1.018 (1.010–1.026)**	**0.711 (0.651–0.771)**	**1.013 (1.004–1.021)**	**0.771 (0.716–0.826)**	**1.011 (1.003–1.020)**	**0.402 (0.341–0.464)**	**0.985 (0.974–0.996)**
Imaging Score	**Epilepsy**		**Return to work**		**Rebleeding**		**DCI**	
	**AUROC (95% CI)**	**Adj. OR (95% CI)**	**AUROC (95% CI)**	**Adj. OR (95% CI)**	**AUROC (95% CI)**	**Adj. OR (95% CI)**	**AUROC (95% CI)**	**Adj. OR (95% CI)**
Modified Fisher	0.592 (0.487–0.698)	1.007 (0.974–1.042)	**0.302 (0.245–0.359)**	0.965 (0.916–1.016)	0.580 (0.485–0.676)	1.016 (0.990–1.044)	**0.643 (0.588–0.698)**	**1.084 (1.043–1.126)**
Claassen	0.601 (0.497–0.706)	1.010 (0.976–1.045)	**0.289 (0.234–0.345)**	0.955 (0.907–1.005)	0.584 (0.489–0.680)	1.018 (0.990–1.046)	**0.651 (0.596–0.705)**	**1.090 (1.049–1.132)**
Hijdra	**0.621 (0.525–0.717)**	1.001 (0.994–1.008)	**0.311 (0.254–0.368)**	**0.986 (0.974–0.998)**	**0.644 (0.551–0.738)**	1.007 (1.001–1.013)	**0.634 (0.580–0.688)**	1.014 (1.004–1.023)
Graeb	**0.632 (0.528–0.735)**	1.020 (0.996–1.044)	**0.292 (0.237–0.348)**	0.984 (0.954–1.014)	0.537 (0.451–0.622)	0.996 (0.985–1.007)	**0.601 (0.545–0.656)**	1.020 (1.000–1.041)
IVH Score	**0.623 (0.519–0.727)**	1.006 (0.997–1.015)	**0.312 (0.255–0.369)**	0.995 (0.982–1.009)	0.519 (0.429–0.609)	0.998 (0.993–1.004)	**0.604 (0.550–0.658)**	1.009 (1.000–1.017)

Multivariable analyses were performed for the primary and secondary endpoints. Values in bold indicate statistically significant associations. Adjusted odds ratios represent the change in odds per one-point increase in the respective imaging score. Adjusted odds ratios represent the change in odds of unfavorable outcome (mRS 4–6) per one-point increase in imaging score; values >1 indicate higher odds, whereas values <1 indicate lower odds of this endpoint. Abbreviations: mRS, modified Rankin scale; CSF shunt, cerebrospinal fluid shunt; DCI, delayed cerebral ischemia, AUROC, area under the curve in receiver operating characteristics analysis; adj. OR, adjusted odds ratio; 95%CI, 95% confidence interval.

## Data Availability

All data supporting the findings of this study are contained within the article.

## Abbreviations

DCI	Delayed cerebral ischemia
SAH	Subarachnoid hemorrhage
ICH	Intracerebral hemorrhage
HH	Hunt and Hess
WFNS	World Federation of Neurosurgical Societies
IVH	Intraventricular hemorrhage
mRS	Modified Rankin scale
CSF	Cerebrospinal fluid
CI	Confidence interval
aOR	Adjusted odds ratio
